# The Efficacy and Safety of Gefapixant in a Phase 3b Trial of Patients with Recent-Onset Chronic Cough

**DOI:** 10.1007/s00408-023-00606-w

**Published:** 2023-03-06

**Authors:** Lorcan McGarvey, Mandel Sher, Yury Grigorievich Shvarts, Susan Lu, Wen-Chi Wu, Ping Xu, Jonathan Schelfhout, Carmen La Rosa, Allison Martin Nguyen, Paul A. Reyfman, Amna Sadaf Afzal

**Affiliations:** 1 Wellcome-Wolfson Institute for Experimental Medicine, School of Medicine, Dentistry & Biomedical Science, Queen’s University Belfast, 97 Lisburn Road, Belfast, BT9 7BL Northern Ireland; 2 Sher Allergy Specialists, LLC, Largo, FL USA; 3 Saratov Medical University, Saratov, Russia; 4 Merck & Co., Inc, Rahway, NJ USA

**Keywords:** P2X3 receptor antagonists, Antitussives, Chronic cough, Refractory chronic cough, Unexplained chronic cough

## Abstract

**Purpose:**

We evaluated gefapixant, a P2X3 receptor antagonist, in participants with recent-onset (≤ 12 months) refractory chronic cough (RCC) or unexplained chronic cough (UCC).

**Methods:**

Participants (≥ 18 years of age; ≥ 40 mm on a 100-mm cough severity visual analog scale [VAS] at screening and randomization) with chronic cough for < 12 months were enrolled in this phase 3b, double-blind, placebo-controlled, parallel group, multicenter study (NCT04193202). Participants were randomized 1:1 to gefapixant 45 mg BID or placebo for 12 weeks with a 2-week follow-up. The primary efficacy endpoint was change from baseline at Week 12 in Leicester Cough Questionnaire (LCQ) total score. Adverse events were monitored and evaluated.

**Results:**

There were 415 participants randomized and treated (mean age 52.5 years; median [range] duration 7.5 [1–12] months): 209 received placebo and 206 received gefapixant 45 mg BID. A statistically significant treatment difference of 0.75 (95% CI: 0.06, 1.44; *p* = 0.034) for gefapixant vs. placebo was observed for change from baseline in LCQ total score at Week 12. The most common AE was dysgeusia (32% gefapixant vs. 3% placebo participants); serious AEs were rare (1.5% gefapixant vs. 1.9% placebo participants).

**Conclusion:**

Gefapixant 45 mg BID demonstrated significantly greater improvement in cough-specific health status from baseline compared to placebo, in participants with recent-onset chronic cough. The most common AEs were related to taste and serious AEs were rare.

**Supplementary Information:**

The online version contains supplementary material available at 10.1007/s00408-023-00606-w.

## Introduction

Patients with chronic cough (cough persisting > 8 weeks) experience considerable burden to health-related quality of life. While chronic cough associated with underlying conditions (e.g., asthma, allergic rhinitis, or gastroesophageal reflux disease) can be successfully treated in some patients, it persists in many patients despite treatment for those underlying conditions (refractory cough; RCC). For other patients, no associated condition is identified despite thorough workup (unexplained chronic cough; UCC). For both groups of patients, there are no licensed treatments, which represents a considerable unmet medical need.

Gefapixant is a P2X3 receptor antagonist with recently confirmed efficacy in two large phase 3 randomized, controlled clinical trials of participants with RCC or UCC who had suffered from chronic cough for many years [1]. Eligibility for enrollment in these Phase 3 trials required a duration of chronic cough for more than 1 year, although among the participants who were enrolled in the trials, the actual mean duration of chronic cough was over 11 years [1, 2]. However, it is unknown if patients with chronic cough lasting less than 1 year may benefit from treatment with gefapixant. The present study assesses the efficacy and safety of gefapixant in participants with RCC or UCC who were considered to have recent-onset chronic cough (ROCC), defined for the purpose of this trial as cough that has persisted for less than 1 year from the onset of chronic cough.

## Methods

This was a Phase 3b, double-blind, randomized, parallel group, placebo-controlled study (NCT04193202, Sponsor Protocol Number 043). The study was approved by local institutional review boards and followed principles of Good Clinical Practice. All participants signed written informed consent.

### Study Design and Participants

Participants were 18 years of age or older with ROCC, defined as a chronic cough (i.e., cough lasting > 8 weeks) with an onset < 12 months prior to the screening visit (i.e., < 14 months after the onset of cough symptoms). Participants’ chronic cough was diagnosed as either RCC or UCC according to the American College of Chest Physicians (ACCP) guidelines [3]. Specific definitions for RCC and UCC can be found in the Supplement. Participants’ self-rated cough severity using a 100-mm visual analog scale [VAS; 0 = no cough, 100 = extremely severe cough] was at least 40 mm at both screening and baseline visits and they had no substantial abnormalities on chest radiograph or computerized tomography (CT) scan of the thorax (within 1 year of study participation and after the onset of cough) contributing to chronic cough.

**Fig. 1:**
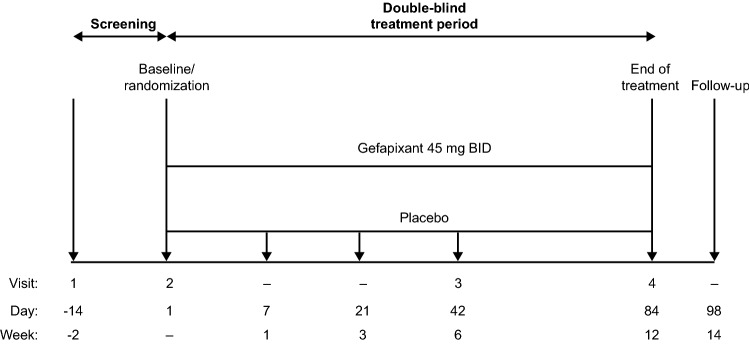
Study design

Major exclusion criteria included active or recent smoking (within 12 months), angiotensin-converting enzyme inhibitor treatment (within 3 months), and FEV1/FVC ratio < 60% within a year prior to study entry. Study procedures are detailed in Fig. 1 and the Supplement.

###  Randomization and Blinding

Eligible participants were allocated to either placebo or gefapixant 45 mg BID in a 1:1 ratio. The randomization was stratified by sex and geographic region and was done by a centralized interactive response technology system. The randomization schedule was computer generated. This study used a double-blind design in which participants and all personnel involved with the conduct and the interpretation of the study were blinded to study treatment.

## Outcome Measures

Our hypothesis was that gefapixant is superior to placebo in improving cough-specific health status in patients with ROCC. The primary efficacy outcome was the Leicester Cough Questionnaire (LCQ) and was analyzed as the total score change from baseline at Week 12. A within-patient 1.3-point increase from baseline in total LCQ is considered to be a clinically meaningful improvement (i.e., responder) [4]. As one of the pre-specified key secondary endpoints in COUGH-2 was a responder analysis for the LCQ endpoint, we also conducted a post hoc analysis to evaluate LCQ responders. Subgroup analyses were conducted for the following baseline factors: sex (male, female), region (North America, Europe, Asia–Pacific, Other), age group (< 60, ≥ 60 years old), and baseline cough severity VAS (< 60 mm, ≥ 60 mm).

The secondary efficacy endpoint was the change from baseline in Cough Severity VAS score at Week 12. We also evaluated the Cough Severity Diary (CSD). For both VAS and CSD, a mean weekly total score was calculated from the daily scores.

The Patient Global Impression of Change (PGIC) and the Work Productivity and Activity Impairment (WPAI) questionnaires were administered and evaluated as exploratory endpoints. Further descriptions of the efficacy outcome measurements are detailed in the Supplement.

Assessments of adverse events (AE) and discontinuations due to AEs were additional secondary objectives in this study. AEs were assessed by clinical evaluation. Other study parameters including vital signs, physical examination, and laboratory safety tests were also evaluated. Investigators evaluated AEs for relationship to study medication, intensity, and seriousness. Taste-related AEs (i.e., ageusia, dysgeusia, hypergeusia, hypogeusia, and taste disorder) were predefined as AEs of special interest.

### Statistical Analysis

The planned sample size was 414 participants (207 per treatment group) for comparing the primary endpoint of LCQ change from baseline in total score at Week 12, assuming a pooled SD of 3.5 points. These assumptions were based on a phase 2 study in participants with RCC or UCC [5]. In this study, the sample size was planned to detect a treatment difference of 1.1 points or more with at least 80% power at an overall one-sided 0.025 alpha level, adjusted for the interim analysis (*α* = 0.001) for strong benefit and the final analysis (*α* = 0.024). This sample size accounted for a 15% attrition rate, targeting for 352 evaluable participants at Week 12.

The primary efficacy analyses were based on the Full Analysis Set (FAS) and were conducted based on the observed data only with no imputation for missing diaries or missing questionnaire items. The pattern of missingness was assumed to be missing at random. The primary efficacy endpoint of this study is the change from baseline in LCQ total score and the primary analysis approach used a longitudinal ANCOVA model. In this model, the response vector consisted of the change from baseline in LCQ total score at each post-baseline visit. The model included factors for intervention group, visit, interaction of treatment by visit, gender, and the baseline LCQ score. The model used all available LCQ data at Weeks 6 and 12. Contrasts were constructed to compare the gefapixant arm to the placebo arm and least squares (LS) mean change from baseline with associated standard errors were calculated for each intervention group. Overall estimated treatment differences (gefapixant − placebo) were assessed along with corresponding *p*-values and 95% CIs. Statistical methodology for secondary, exploratory, subgroup, and post hoc efficacy analyses is shown in the Supplement.

## Results

### Participants

**Fig. 2:**
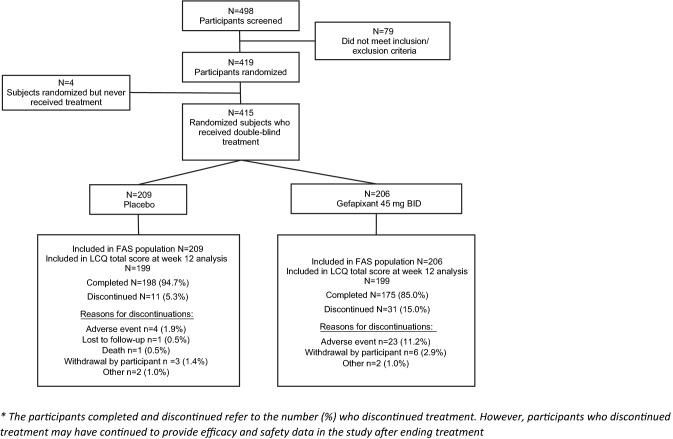
CONSORT diagram

Of 498 screened participants, 419 were randomized and 415 received treatment (*n* = 209 in the placebo group and *n* = 206 in the gefapixant 45 mg BID group). There were 11 (5.3%) participants in the placebo arm and 31 (15.0%) participants in the gefapixant arm who discontinued treatment (Fig. 2). Of the 415 treated participants, 268 (65%) were female, 300 (72%) were white, and the mean age was 52.5 years old. The primary diagnosis of participants was RCC [*n* = 294 (71%)] or UCC [*n* = 121 (29%)]. (Table 1). The mean duration of chronic cough was 7.2 months, with a median duration of 7.5 months. The distribution of duration for all patients is illustrated in Supplemental Fig. 1.

**Table 1:** Baseline participant characteristics

	Placebo *N* = 209	Gefapixant 45 mg BID *N* = 206
	*n* (%)	*n* (%)
Female	134 (64.1)	134 (65.0)
Age (yr), Mean ± SD	52.5 ± 13.8	52.5 ± 13.8
< 60	135 (64.6)	130 (63.1)
≥ 60	74 (35.4)	76 (36.9)
Median (range)	55.0 (18 to 83)	54.0 (18 to 81)
Race		
White	151 (72.2)	149 (72.3)
Multiple	29 (13.9)	29 (14.1)
American Indian or Alaska Native	27 (12.9)	22 (10.7)
Asian	2 (1.0)	3 (1.5)
Black or African American	0 (0.0)	3 (1.5)
Hispanic or Latino	75 (35.9)	71 (34.5)
Region		
Europe	123 (58.9)	122 (59.2)
Other*	74 (35.4)	72 (35.0)
North America	11 (5.3)	10 (4.9)
Asia–Pacific	1 (0.5)	2 (1.0)
Primary diagnosis		
Refractory chronic cough	144 (68.9)	150 (72.8)
Unexplained chronic cough	65 (31.1)	56 (27.2)
Most common comorbid cough-associated conditions**		
Asthma	82 (39.2)	88 (42.7)
Gastroesophageal reflux disease	61 (29.2)	63 (30.6)
Allergic rhinitis	34 (16.3)	35 (17.0)
Chronic gastritis	23 (11.0)	35 (17.0)
Duration of chronic cough with diagnosis (months)		
Mean ± SD, months	7.2 ± 2.7	7.3 ± 2.8
Median, months	7.0	8.0
Range, months	2 to 12	1 to 12
Baseline LCQ total score		
Mean (SD)	11.30 (2.80)	10.82 (3.08)
Baseline mean weekly cough severity VAS (mm)		
< 60	80 (38.3)	73 (35.4)
≥ 60	129 (61.7)	133 (64.6)
Mean ± SD	66.2 ± 14.9	67.2 ± 14.9
Median (range)	64.7 (22.3 to 100.0)	65.8 (27.7 to 100.0)

*Other includes countries in Central and South America; ** Participants may have had more than one comorbid cough-associated condition

### Efficacy Outcomes

**Fig. 3:**
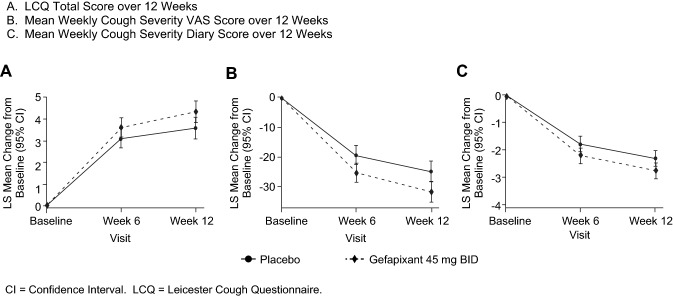
LS mean change from baseline in efficacy endpoints over 12 Weeks

There were improvements in total score for the primary endpoint (change from baseline in LCQ total score at week 12) for both treatment groups. The model-based mean changes from baseline (95% CI) were 3.59 (3.09, 4.09) for placebo and 4.34 (3.84, 4.83) for gefapixant. This resulted in an estimated difference of 0.75 (0.06, 1.44) indicating that gefapixant 45 mg BID was superior to placebo in improving LCQ total score at Week 12 (*p* = 0.034) (Fig. 3).

Results for the post hoc analysis revealed a larger percentage of participants in the gefapixant 45 mg BID arm achieved a 1.3-point improvement from baseline in LCQ total score compared with those in the placebo arm (80.6% vs. 67.4%) with an estimated difference of 13.19% (95% CI: 3.46, 22.81) and an estimated odds ratio of 2.01 (95% CI: 1.21, 3.32).

The subgroup analysis according to baseline characteristics (sex, region, age group, and baseline cough severity VAS scores) demonstrated similar improvement in LCQ total score among each subgroup (Supplemental Fig. 2).

A treatment difference of − 6.92 (95% CI: − 11.88, − 1.97) based on LS mean change from baseline in mean weekly cough severity VAS score at Week 12 and − 0.47 (95% CI: − 0.88, − 0.06) change from baseline for CSD total score were observed, showing improvement with gefapixant vs. placebo in both endpoints (Table 2).

**Table 2:** Summary of patient-reported outcomes assessing treatment efficacy

	Placebo	Gefapixant 45 mg BID
Pre-specified analyses		
*Primary Endpoint*		
LCQ total score at week 12		
Number of Participants Evaluated^†^	199	199
Model-Based Mean Change from Baseline (95% CI)*	3.59 (3.09, 4.09)	4.34 (3.84, 4.83)
Estimated Difference (95% CI) from Placebo, p-value		0.75 (0.06, 1.44), *p=0.034*
*Secondary and exploratory endpoints*		
Mean weekly cough severity VAS score at week 12		
Number of Participants Evaluated	205	201
Model-Based Mean Change from Baseline (95% CI)*	− 24.87 (− 28.41, − 21.32)	− 31.79 (− 35.37, − 28.20)
Estimated Difference (95% CI) from Placebo		− 6.92 (− 11.88, − 1.97)
Mean weekly CSD total score at week 12		
Number of Participants Evaluated	205	201
Model-Based Mean Change from Baseline (95% CI)*	− 2.32 (− 2.61, − 2.03)	− 2.79 (− 3.08, − 2.49)
Estimated Difference (95% CI) from Placebo		− 0.47 (− 0.88, − 0.06)

Post hoc analysis		
Participants with ≥ 1.3-point increase from baseline in LCQ total score at week 12		
Number of Participants Evaluated	208	202
Percent Responders	67.4% (Placebo)	80.6%
Estimated Difference (95% CI) from Placebo **		13.19 (3.46, 22.81)
Estimated Odds Ratio vs. Placebo (95% CI) *		2.01 (1.21, 3.32)

*Based on the Longitudinal Analysis of Covariance Model, consisting of the change from baseline in mean weekly cough severity VAS score at each post-baseline visit (up to Week 12) as response. The model includes terms for treatment group (gefapixant 45 mg BID and Placebo), visit (Weeks 6 and 12), the interaction of treatment by visit, gender, and the baseline mean weekly cough severity VAS score. The unstructured covariance matrix is used to model the correlation among repeated measurements

**Based on the logistic regression model. The covariates include treatment, visit, the interaction of treatment by visit, gender, and the baseline LCQ total score

^†^Number of participants based on FAS population in participants with non-missing values at both baseline and Week 12

There were a greater proportion of participants treated with gefapixant 45 mg BID who reported improvement on the PGIC compared to those in the placebo arm. The estimated treatment difference for the responder definition was 8.8% (95% CI: 0.50, 17.11). The greatest difference between gefapixant 45 mg BID and placebo on the categories used to define responders was in those who reported feeling “much better” (33.8% vs. 19.6%) (Supplemental Fig. 3).

Additionally, the WPAI assessment indicated that the gefapixant arm had greater work productivity and activity scores, with notable differences in the categories of impairment while working due to cough and overall work impairment due to cough items. (Supplemental Fig. 4).

### Summary of Adverse Events

The percentages of participants with AEs were 43.1% in the placebo group and 65.5% in the gefapixant 45 mg BID group. The most common AEs were related to taste and were more commonly reported by participants on gefapixant. The overall incidence of serious AEs was < 2% and similar between placebo and gefapixant. There was one death, which occurred in the placebo group. Discontinuations due to AEs were higher in the gefapixant 45 mg BID group (Table 3).

**Table 3:** Summary of adverse events

	Placebo *N* = 209	Gefapixant 45 mg BID *N* = 206
	*n* (%)	*n* (%)
≥ 1 AEs	90 (43.1)	135 (65.5)
Drug-related AEs	20 (9.6)	112 (54.4)
Serious AEs	4 (1.9)	3 (1.5)
Serious drug-related AEs	0 (0.0)	0 (0.0)
Deaths	1 (0.5)	0 (0.0)
Due to a drug-related AE	0 (0.0)	0 (0.0)
Discontinued drug due to an AE	4 (1.9)	23 (11.2)
Due to a drug-related AE	1 (0.5)	18 (8.7)
Due to a serious AE	1 (0.5)	0 (0.0)
Due to a serious drug-related AE	0 (0.0)	0 (0.0)
Most common AEs		
Ageusia	0	24 (11.7)
Dysgeusia	7 (3.3)	66 (32.0)
Headache	14 (6.7)	11 (5.3)
Hypogeusia	1 (0.5)	22 (10.7)

## Discussion

The findings from this study confirm our hypothesis that, compared to placebo, gefapixant significantly improves cough-related quality of life in patients with ROCC, consistent with previous Phase 3 data from COUGH-1 and COUGH-2 in RCC and UCC participants who had chronic cough of longer duration^1^. The demographics of participants randomized in this study were broadly similar to those of COUGH-1 and COUGH-2 and consistent with previous chronic cough clinical trials as well as data reported from observational studies in the general population [1, 6]. In this trial, the mean age of participants was approximately 7 years younger and duration of chronic cough was considerably shorter, as expected, compared with COUGH-1 and COUGH-2 (i.e., mean of ~7 months vs. ~11 years) [1].

Our results suggest that treatment with gefapixant 45 mg BID improves cough-specific health status to a greater extent than placebo in patients with RCC or UCC who are younger and have had chronic cough for a shorter period of time than previously studied. The majority of the PROs improved by 6 weeks, when the first measurements were collected, and benefits continued to improve over the 12-week study.

While a statistically significant improvement in the in LCQ total score was observed with gefapixant compared to placebo, it should be acknowledged that the estimated between-group difference was numerically small (0.75), due primarily to the large improvement in LCQ observed for both the gefapixant and placebo groups. However, results from the post hoc analysis to determine the proportion of participants reporting a clinically meaningful improvement in LCQ total score (i.e., a > 1.3-point improvement in total LCQ score) showed that a greater proportion of participants randomized to gefapixant 45 mg BID were LCQ responders compared to those in the placebo group. This finding that treatment with gefapixant conferred a greater likelihood of improvement than placebo is consistent with the findings in COUGH-2 [1].

The results of the additional PROs assessed in this study, the change in mean cough severity VAS score, and mean weekly CSD total score were consistent with the primary endpoint and supportive of our hypothesis that gefapixant is superior to placebo in the treatment of RCC and UCC in patients with ROCC. The results from the PGIC, which provides an impression of how patients generally feel regarding treatment of their cough, demonstrated improvement in a higher proportion of patients receiving gefapixant compared to placebo with greatest differences between treatments noted in the category of those feeling “much better.” Results from the WPAI suggested improvement in work productivity and less activity impairment for gefapixant compared to placebo.

We did not identify any single baseline characteristic to be more associated with mean change in LCQ from baseline, which is consistent with an analysis of baseline characteristics in relation to 24-h cough frequency in the COUGH-1 and COUGH-2 trials. Similar to the results presented here for ROCC, no baseline characteristics were found to be associated with reduced or greater efficacy in COUGH-1 or COUGH-2 in participants with RCC or UCC of longer baseline duration [1].

Results from this trial are consistent with previous research with gefapixant in that the most commonly reported AEs were taste related [1, 5, 7]. However, serious AEs occurred in a similar proportion among both the gefapixant- and placebo-treated participants.

The large placebo response observed in the current study is consistent with other gefapixant trials [1, 5] and studies of chronic cough [8, 9]. As with gefapixant, improvement in health status with placebo was evident at the first study time point (6 weeks) and continued to the end of study at 12 weeks. It is not clear whether such improvements would be maintained over longer periods of time in the ROCC group of patients with RCC or UCC. There is likely a multifactorial cause for the placebo responses that have been observed consistently in trials of treatments for chronic cough. One factor may be anticipation of a treatment that has shown positive results in a condition that has no approved treatment. However, it is unlikely to be the sole contributor based on the magnitude of the response observed. Such dramatic improvements in health status among patients with chronic cough have not been reported in other settings in the absence of an active treatment. Another contributor to the observed phenomenon is that cough is a reflex that can be under voluntary control with higher brain involvement [9, 10]. Higher brain involvement has been suggested as a factor for placebo responses in other respiratory conditions [11–14] and has been observed as a factor in cough challenge studies [15]. Nonetheless, although a large placebo response was observed in this trial, participants in this trial experienced important improvements in cough-specific health status with gefapixant vs. placebo.

There were limitations in this study that should be acknowledged. There was no objective cough measure to compare with the primary endpoints of Phase 2b and Phase 3 studies. There was also no pre-specified analysis plan to reliably determine the timing of onset of efficacy. Additionally, while we demonstrated that intervention with gefapixant earlier in the natural history of RCC or UCC was successful in terms of improvements in cough-related health status, longer-term benefits of earlier treatment have yet to be evaluated. Nevertheless, results from this study demonstrate favorable effects on multiple PROs that are consistent with results observed in the larger, Phase 3 studies [1].

In summary, treatment with gefapixant 45 mg BID provided significantly greater improvements in cough-specific health status than placebo in patients with ROCC. Patients treated with gefapixant were also more likely to report clinically meaningful improvements in cough-specific health status and larger reductions in patient-reported measures of cough severity compared to placebo. The most common AEs were related to taste, consistent with previous studies of gefapixant.[1, 5]. Serious AEs with gefapixant 45 mg BID were uncommon and occurred in a similar proportion of participants as those on placebo. These data indicate a favorable benefit/risk profile for gefapixant in the treatment of individuals with RCC or UCC with a diagnosis of chronic cough for less than a year.

## Supplementary Information

Below is the link to the electronic supplementary material.

Supplementary file1 (DOCX 556 KB)
