# Supplementary material for: The Efficacy and Safety of Gefapixant in a Phase 3b Trial of Patients with Recent-Onset Chronic Cough
Source: Lung. 2023 Mar 6;201(2):111–8. doi: 10.1007/s00408-023-00606-w (PMC10115701; doi:10.1007/s00408-023-00606-w)
Supplement: Supplementary file 1 — Supplementary file1 (DOCX 556 KB) [file 408_2023_606_MOESM1_ESM.docx]

**Supplement**

Definition of RCC and UCC

RCC was defined as cough that persisted in participants with a comorbid condition related to the cough (e.g., reflux disease, asthma, or allergic rhinitis) who received appropriate diagnostic work-up and therapy (for at least 2 months for known conditions) according to ACCP guidelines. UCC was defined as chronic cough in participants who had a clinical evaluation per ACCP guidelines that did not suggest a comorbid condition related to the cough.

Study Procedures

Participants were screened over 14 days and eligible participants were randomized to gefapixant 45 mg BID or placebo at baseline. Following screening, the study intervention period, including randomization, was approximately 84 days (i.e., 12 weeks), with a 14-day follow-up period during which adverse events were monitored. The study was comprised of 4 study visits and 3 scheduled telephone calls. Visit 1 was Screening from -2 weeks to -1 week, Visit 2 was Baseline/Randomization/Day 1, Visit 3 was Week 6, Visit 4 was Week 12, and Telephone Calls were on Day 7 and Day 21, and a Safety Follow-up Telephone Call was at Week 14 (Figure 1).

An external data monitoring committee (DMC) monitored efficacy and safety. One interim analysis was planned when approximately 40% of the total randomized participants either completed or discontinued the study.

Participants were trained on the use of an e-Diary for completing patient-reported outcomes (PRO) at Visit 1. The Cough Severity VAS and CSD were completed each evening between visits. At the clinic visits, participants completed the Cough Severity VAS and LCQ. Participants who discontinued study intervention early continued to be monitored and were encouraged to continue to complete PROs in their e-Diaries through the end of the study.

Statistical methods for additional efficacy analyses

Subgroup analyses utilized a longitudinal ANCOVA model similar to the one used for the primary efficacy endpoint. For each subgroup, summary statistics including means and their 95% CIs were calculated for each intervention group at Week 12. For the subgroups with 15% or more of the FAS population across both intervention groups, the mean treatment differences (gefapixant - placebo) and their 95% CIs were also calculated at Week 12. For the post-hoc analysis of participants with ≥1.3-point increase from baseline in total LCQ score, treatment differences (gefapixant – placebo) and 95% CIs were calculated using a logistic regression model for repeated measures with covariates that include treatment, visit, the interaction of treatment by visit, gender, and the baseline LCQ total score.

The Cough Severity VAS was analyzed using a similar longitudinal ANCOVA model as used for the primary efficacy analysis. The model includes terms for intervention group, visit, interaction of treatment by visit, gender, and baseline score. A participant was considered for inclusion in the assessment of an efficacy endpoint if the Cough Severity VAS was completed on at least 4 days during the 7-day period prior to a post-baseline visit.

The analysis of the exploratory endpoint of mean weekly change in the CSD was also based on the longitudinal ANCOVA model and consisted of the change from baseline in mean weekly CSD score at each post-baseline visit (up to Week 12) as response. The model includes terms for treatment group (gefapixant 45 mg BID and placebo), visit (Weeks 6 and 12), the interaction of treatment by visit, gender, and the baseline mean weekly CSD score. The unstructured covariance matrix was used to model the correlation among repeated measurements. The analysis of treatment responders on gefapixant vs. placebo on the exploratory endpoint of PGIC was based on the stratified Miettinen and Nurminen method with gender and region as stratification factors, after nonresponder imputation.

Efficacy Outcomes

| Leicester Cough Questionnaire (LCQ) | LCQ is a validated, 19-item, cough-specific health-related quality-of-life (HRQoL) questionnaire that contains three domains (physical, psychological, and social). Each domain score is calculated as the mean score of the items within the domain, with a range from 1 to 7. The LCQ total score is the sum of the 3 domains, with a range from 3 to 21. Higher scores indicate better HRQoL^12^. |
| --- | --- |
| Cough Severity Visual Analog Scale (VAS) | The VAS is a single-item question asking the participant to rate the severity of their cough “today” using a 100-mm VAS anchored with “No Cough” at 0 and “Extremely Severe Cough” at 100. |
| Cough Severity Diary (CSD) | The CSD is a validated, 7-item, cough-specific patient-reported outcomes (PRO) measure with a recall period of “today.” The 7 items evaluate frequency of cough (3 items), intensity of cough (2 items), and disruption due to cough (2 items). Each item is scored from 0 (best) to 10 (worst). The total is the sum of these items. |
| Patient Global Impression of Change (PGIC) | The PGIC is a 2-part measure asking the participant to rate the change in their cough symptoms compared to the start of the study, with response options ranging from “much better” to “much worse” (7-point scale). Based on the response to this initial question, the participant was asked a follow-up question to evaluate the meaningfulness of the improvement or worsening, with response options of ‘yes’ or ‘no’ indicating whether the improvement or worsening was important or not. |
| Work Productivity and Activity Impairment (WPAI) | The WPAI questionnaire yields 4 types of scores as follows: (1) absenteeism (work time missed); (2) presenteeism (impairment at work/reduced on-the-job effectiveness); (3) work productivity loss (overall work impairment/absenteeism plus presenteeism); and (4) activity impairment. Participants were asked to indicate if they are currently employed and to respond to the following questions referring to “the past 7 days”: work hours missed due to health problems, work hours missed for other reasons, hours actually worked, the degree to which their health has affected productivity while working, and the degree to which their health affected productivity in regular unpaid activities. |

Supplemental Figure 1 – Distribution of Participants According to Baseline Duration of Chronic Cough

|  |  |  |
| --- | --- | --- |

Supplemental Figure 2 – LCQ Total Score Subgroup Analysis





*Other includes countries in Central and South America

Supplemental Figure 3 – Analysis of PGIC at Week 12





Improvement Responder = participants who responded with “Much Better”, “Better”, or “A Little Better”

Supplemental Figure 4 - Analysis of WPAI Score at Week 12





*The ‘Percent Work Time Missed’, ‘Percent Impairment While Working’, and ‘Percent Overall Work Impairment’ items were answered only by participants who work for pay while the ‘Percent Activity Impairment’ item was answered by all participants.*
